# Cognitive and Neuropsychiatric Effects of 40 Hz tACS Simultaneously with Cognitive Exercises for Dementia: A Randomized, Crossover, Double-Blind, Sham-Controlled Study

**DOI:** 10.3390/medicina61040757

**Published:** 2025-04-19

**Authors:** Maria Anabel Uehara, Sumeet Kalia, Mari Garcia Campuzano, Mohammad Jafari-Jozani, Brian Lithgow, Zahra Moussavi

**Affiliations:** 1 Biomedical Engineering, University of Manitoba, Winnipeg, MB R3T 5V6, Canada; zahra.moussavi@umanitoba.ca; 2 Department of Statistics, University of Manitoba, Winnipeg, MB R3T 5V6, Canada; sumeet.kalia@umanitoba.ca (S.K.); m_jafari_jozani@umanitoba.ca (M.J.-J.); 3 Department of Electrical and Computer Engineering, University of Manitoba, Winnipeg, MB R3T 5V6, Canada; maritere.garciacampuzano@umanitoba.ca; 4 Riverview Health Centre, Winnipeg, MB R3L 2P4, Canada; brian.lithgow@umanitoba.ca; 5 Monash Alfred Psychiatry Research Centre, Melbourne, VIC 3004, Australia

**Keywords:** transcranial alternating current stimulation (tACS), cognitive exercise, dementia, non-invasive brain stimulation, crossover, clinical trial, ADAS-Cog, neuromodulation

## Abstract

*Background and Objectives*: Transcranial alternating current stimulation (tACS) at 40 Hz has shown potential to enhance cognitive function. However, research on its combination with cognitive exercises, particularly its long-term effects in a dementia population, remains limited. This study investigated the effects of 40 Hz tACS paired with simultaneous cognitive exercises on cognition, neuropsychiatric symptoms, and the depression status of individuals with dementia in a sham-controlled, double-blind crossover design. *Materials and Methods*: A total of 42 participants with dementia were randomized into two groups: (1) the R1S2 group received 40 Hz real tACS with cognitive exercises, followed by a ≥8-week washout period, and then sham tACS with cognitive exercises; (2) the S1R2 group received the reversed sequence. tACS was applied at 1.5 mA peak-to-peak with electrodes over the left dorsolateral prefrontal cortex and contralateral supraorbital area. Participants received two 30 min stimulation sessions per day, 5 days per week, for 4 consecutive weeks, paired with cognitive exercises using the MindTriggers app (2.9.1). The primary outcome was the Alzheimer’s Disease Assessment Scale–Cognitive Subscale (ADAS-Cog) and the secondary outcomes included the Montgomery–Åsberg Depression Rating Scale (MADRS) and the Neuropsychiatric Inventory Questionnaire (NPI-Q). All outcome measures were assessed before and after each treatment block. *Results*: Real tACS paired with cognitive exercises significantly improved ADAS-Cog scores post-treatment compared to pre-treatment (*p*-value = 0.019), whereas sham tACS did not. Furthermore, real tACS produced significant long-term improvements approximately 2–3 months post-treatment in ADAS-Cog scores compared to sham (*p*-value = 0.048). Both real (*p*-value = 0.003) and sham (*p*-value = 0.015) tACS significantly reduced NPI-Q scores post-treatment. MADRS scores significantly improved (*p*-value = 0.007) post-treatment for real tACS but not sham. *Conclusions*: The 40 Hz tACS paired with cognitive exercises improves cognition, neuropsychiatric symptoms, and depression post-treatment in dementia, with sustained cognitive effects. The findings highlight its potential as a non-invasive therapeutic intervention for dementia.

## 1. Introduction

Dementia affects over 55 million individuals globally, and its prevalence is expected to increase further with aging populations [1]. Neurodegenerative dementia including Alzheimer’s disease (AD) as its most common type, is a progressive condition characterized by memory loss, difficulties with problem-solving, language deficits, and challenges in performing daily activities [2]. Despite significant advancements in research, there remains no cure for dementia, highlighting an urgent need for innovative therapeutic approaches such as non-invasive brain stimulation and cognitive training to mitigate cognitive decline, improve the quality of life, slow the progress of the condition, and avoid institutionalization. In recent years, non-invasive brain stimulation techniques, such as transcranial alternating current stimulation (tACS) and cognitive training, have been investigated for their potential therapeutic effects in slowing down the progression of dementia and improving cognitive function. This paper presents the results of gamma-tACS (at 40 Hz) paired with simultaneous cognitive exercises as a treatment for individuals with dementia (the majority (62%) with AD) in a double-blind, sham-controlled crossover design study.

tACS has gained attention for its ability to modulate brain activity and target specific neural oscillations associated with cognition [3]. It is a non-invasive, relatively inexpensive and safe brain stimulation technology that is easy to administer; it applies low-amplitude oscillating electrical currents through scalp electrodes to entrain neural oscillatory activity in targeted frequency bands [4]. Neural oscillations play a critical role in regulating cognitive processes, and specific frequency bands have been linked to distinct cognitive domains. Specifically, the gamma band (~30–100 Hz) is associated with higher cognitive functions such as learning, episodic and working memory, and attention [5]. Given that dementia is characterized by disruptions in neural oscillations critical for memory and attention, tACS offers a targeted therapeutic approach to restore these oscillatory patterns and address associated cognitive impairments.

Our research team previously conducted two pilot studies to investigate the cognitive effects of daily tutored cognitive exercises without or with simultaneous gamma-tACS (40 Hz) with cognitive exercises. The first was a study of cognitive exercise for 8 weeks, 3 days/week, on 24 healthy older adults placed in either the cognitive training (*n* = 13) group or the control (*n* = 11) group [6]. The results showed a significant improvement in Wechsler Memory Scale (WMS) scores from baseline to post-treatment in the cognitive training group compared to the control group. The second was a treatment study on 28 individuals with AD in two groups receiving tutored cognitive exercises with tACS (*n* = 19) and without (*n* = 9) tACS for 4 weeks, 5 days/week [7,8]. Gamma (40 Hz)-tACS was administered at 1.5 mA peak-to-peak for 30 min over the left dorsolateral prefrontal cortex (DLPFC), with the reference electrode placed over the contralateral supraorbital area. Treatments were delivered in two 30 min sessions daily, 5 days per week, for 4 weeks. Results showed that both groups revealed statistically significant improvements in WMS scores from baseline to post-treatment; however, only the tACS with cognitive training group showed a marginally significant trend towards sustained improvement at the 1-month follow-up. While these findings suggest potential cognitive benefits of pairing 40 Hz tACS with cognitive exercises, these studies [7,8] lacked a control group with sham tACS.

Beyond our research group, several studies have explored the efficacy of applying multiple sessions of tACS in populations with dementia and MCI as a potential treatment. One sham-controlled study applied 40 Hz tACS at 2 mA over the bilateral temporal lobes to individuals with AD (*n* = 23 tACS, *n* = 27 sham) for 20 min per session, 5 sessions per week, over 6 weeks [9]. The tACS group showed significant improvements in both Mini-Mental State Examination (MMSE) and Alzheimer’s Disease Assessment Scale–Cognitive Subscale (ADAS-Cog) scores immediately following the intervention. However, while MMSE improvements remained significant at the 12-week follow-up, ADAS-Cog scores regressed to baseline, highlighting the potential short-term cognitive benefits of tACS that may require ongoing treatment for sustained effects [9]. Conversely, another study found sustained benefits for a home-based tACS intervention lasting up to 2–3 months [10]. In that single-arm study (*n* = 8, AD), participants received 40 Hz tACS over the left angular gyrus for 20 min per session, 5–7 times per week, for 14 weeks. Memory performance, as measured by the Memory Index Score, improved after the treatment period with sustained benefits observed even during the hiatus phase. However, statistical analyses were not reported, limiting the ability to draw definitive conclusions about the reliability of these effects [10]. A separate study explored different tACS montages by applying 40 Hz stimulation over unilateral temporo-frontal or bitemporal lobes in a small cohort of AD participants (*n* = 15) [11]. Participants received 1 h sessions 5 days per week for either 2 or 4 weeks. Arterial spin labeling magnetic resonance imaging revealed a significant increase in blood perfusion in the bilateral temporal lobes following tACS treatment [11]. Moreover, these perfusion changes correlated positively with improvements in episodic memory and gamma-band spectral power, further supporting the potential of tACS to modulate both neurophysiological and cognitive outcomes in AD [11]. Together, these studies provide growing evidence that 40 Hz tACS can enhance cognitive performance in individuals with MCI and AD. However, methodological variations, including differences in stimulation duration, cortical targets, and study designs, highlight the need for further investigation. While these studies demonstrate cognitive improvements following gamma-tACS, the underlying neural mechanisms remain an area of active research.

The exact mechanism of tACS is still not entirely understood [12]. However, through different approaches including in vivo and in vitro animal studies, the main mechanisms for tACS include the modulation of the neuronal membrane potential, the entrainment of neural oscillations, and spike-timing dependent plasticity [13,14]. At the cellular level, tACS alters the transmembrane potential of neurons by synchronizing with the stimulation, with even small changes capable of affecting the firing rate of active neurons [14]. By delivering oscillating electrical currents, tACS can synchronize brain activity at specific frequencies, such as the gamma band, which is associated with higher cognitive functions like memory processing and attention [5]. This synchronization facilitates communication between brain regions, potentially improving memory consolidation and cognitive performance [15]. Additionally, gamma-tACS may exert therapeutic effects through several mechanisms, such as reducing phosphorylated tau deposition [16], enhancing synaptic plasticity and cognitive function [4], increasing blood oxygenation to specific brain regions when applying at 40 Hz [11,17], and improving intracortical connectivity [18]. These findings suggest that tACS may provide multifaceted benefits by targeting both neuronal and pathological processes underlying dementia.

As another non-invasive therapeutic technologies, cognitive training programs are designed to build cognitive reserve to maintain cognitive function by engaging neural networks through structured exercises [6,19,20]. Previous research indicates that multiple, regimented brain exercise sessions significantly enhance cognitive function in healthy older adults [6,21,22]. One long-term study demonstrated that consistent cognitive training not only improves cognitive performance but also reduces dementia risk over a 10-year follow-up period compared to an untreated control group [23]. Additionally, two studies reported that computer-based cognitive training programs effectively improve impaired cognitive function in early AD [24,25]. However, these benefits were mostly observed in the trained tasks, with limited transfer to untrained tasks, potentially due to unsupervised training [22,23,25,26,27]. Studies suggest that structured interventions, where a treatment administrator guides participants through cognitive exercises daily, show broader improvements in daily life beyond the practiced exercises [6,7,8,28,29]. This approach ensures personalized feedback and sustained participant engagement, optimizing the benefits of cognitive training.

There were several shortcomings in previous studies, including having a small sample size (n < 30) or the lack of a sham-controlled condition to account for placebo effects, and the absence of randomization in group assignments. This study aimed to address these limitations by implementing a sham-controlled, double-blind crossover design to control for placebo effects and minimize inter-subject variability by allowing participants to be their own controls. The goal was to investigate the immediate and longer-term cognitive effects of pairing 40 Hz tACS and cognitive exercises in individuals with dementia.

## 2. Materials and Methods

### 2.1. Study Design and Participants

This was a sham-controlled, double-blind crossover design clinical trial to investigate the effects of tACS paired with cognitive exercises conducted at Riverview Health Centre. The crossover design was selected to control for between-subject variability and maximize statistical power given the limited pool of eligible participants for recruitment, thus allowing each individual to serve as their own control [30]. The study protocol published in [31] details the full methodological framework, including the sample size calculation, which estimated that 60 participants would be enrolled in either the R1S2 or S1R2 group. Participants were randomly assigned to one of two groups, R1S2 or S1R2; the stratification parameters were age (above or below 70 years), sex (male or female), and cognitive level, which was measured using the Montreal Cognitive Assessment (MoCA) (above or below 18 MoCA score) to ensure balanced key demographic characteristics. Randomization was conducted using the PROC PLAN procedure from SAS to generate the groups [32]. The team member who made the database also wrote the code to generate the randomization.

**Figure 1:**
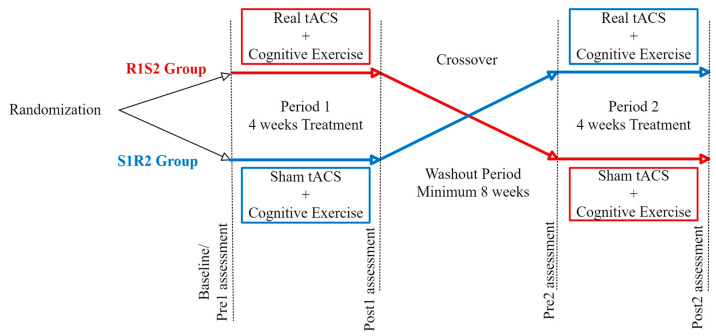
Schematic representation of the crossover study design.

Each group received two 4-week blocks of treatment; the R1S2 group received real tACS in period 1 and sham in period 2, while the S1R2 group received sham first and real second. Both real and sham tACS treatments were paired with cognitive exercises using the MindTriggers app [33]. The two treatment blocks were separated by at least 8 weeks. Figure 1 shows the schematic of the crossover design.

The eligibility criteria for enrolling individuals into this study were as follows:

- Age between 50 and 95 years;
- A MoCA score between 5 and 25;
- Being on a stable dose of dementia-related medication or no medication 3 months prior to the study;
- The ability to read, write, and speak English or Spanish fluently;

Exclusion criteria included were as follows:

- A diagnosis of Parkinson’s disease, Parkinsonian dementia, Huntington’s disease, significant aphasia, intellectual disability, severe depression or anxiety, bipolar disorder, schizophrenia, or other major mood disorders;
- A history of epileptic seizures or epilepsy;
- Impaired vision or hearing severe enough to impair performance in cognitive tests;
- Current substance abuse disorder;
- Currently participating in another therapeutic study for dementia;
- Plans to change their dementia-related medication during the study’s period.

Participants were monitored closely to ensure the adherence of participants to the protocol.

Potential participants were recruited from previous studies or referrals from family physicians, geriatricians, neurologists, and psychiatrists, who also provided the participants’ diagnostic reports. Individuals were interviewed to determine if they met the inclusion and exclusion criteria including the MoCA [34]. All participants, along with a family member or legal guardian, were asked to sign an informed consent form (approved by the Biomedical Research Ethics Board of the University of Manitoba) prior to enrollment. Before the baseline assessment, participants were assessed for their ability to tolerate tACS. If the participant was unable to tolerate tACS, they were offered the option to only perform cognitive exercises, and their performance data were not included in the study analysis presented in this paper. Lastly, each participant was assigned an anonymized code for data recording to ensure confidentiality. Enrollment into the study and assignment of the group was completed by the study coordinator (M.G.C.) by using the computerized randomization code.

### 2.2. Intervention

The treatment protocol for both real and sham tACS was 4 consecutive weeks, 5 days per week (excluding weekends), with two 30 min stimulation paired with cognitive training sessions per day with a 30 min break in between, all in-person. The tACS was administered using the Soterix Medical Stimulator tES (New York, USA) machine with the following parameters:

- Waveform: bipolar sinusoid;
- Frequency: 40 Hz;
- Current Intensity: 0.75 mA amplitude (1.5 mA peak-to-peak).

Sham tACS using the Soterix device administers an initial increasing ramp up of current intensity for 30 s and ramp down to 0 mA for another 30 s and remains at 0 mA until the last minute of stimulation where the current again ramps up and ramps down within 1 min.

Sponge electrodes soaked in saline were placed over the dorsolateral prefrontal cortex (F3 on the 10–20 electroencephalogram systems) and contralateral supraorbital area. The treatment providers were trained to ensure the electrodes had good contact with the scalp by checking the readings on the Soterix machine. They were also asked to record any side effects or adverse effects such as discomfort, sensitivity, burning, fatigue, headaches, dizziness, disorientation, itching/tingling, pain, and any other mentioned sensations they observed, or mentioned by the participant throughout the session.

Participants were instructed and helped by the treatment administrators to perform cognitive exercises using the MindTriggers app while receiving the real or sham tACS [33] on an iPad. The cognitive exercises that every participant played at every treatment session included spatial cognition, associative memory, visual memory, image–word association, categorization, and memorize and rewrite sentences.

### 2.3. Assessments and Outcome Measures

Participants were assessed 3–7 days before the first treatment of each treatment block and 3–7 days after the last treatment of each treatment block by an assessor who was blinded to the treatment type (real or sham). The primary outcome measure was the changes in ADAS-Cog scores, a widely recognized tool for assessing cognitive function in individuals with AD or dementia. The ADAS-Cog consists of 11 tasks that evaluate multiple cognitive domains including memory, language, orientation, and praxis, making it a comprehensive measure for monitoring therapeutic and intervention outcomes [35]. The ADAS-Cog score ranges from 0 to 70, with lower scores indicating better cognitive performance and higher scores reflecting greater cognitive impairment.

The secondary outcome measures were the changes in Neuropsychiatric Inventory Questionnaire (NPI-Q) [36] and the Montgomery–Åsberg Depression Rating Scale (MADRS) [37]. The NPI-Q assesses the severity of neuropsychiatric symptoms over the past month using 12 questions. Symptom severity is rated on a scale from 1 (mild) to 3 (severe), with a total severity score ranging from 0 to 36. The version used in this study does not include caregiver burden. MADRS assesses depression severity across 10 items, and each item is rated on a scale from 0 to 6. Thus, the total score ranges from 0 (no depression) to 60 (severe depression).

### 2.4. Statistical Analysis

The baseline characteristics with continuous data (age, MoCA, MADRS, ADAS-Cog, and NPI-Q) were tested for any significant difference between the R1S2 and S1R2 groups using independent *t*-tests or their non-parametric equivalent if the assumptions of normality and homogeneity of variance were not satisfied. Categorical characteristics (sex, handedness, and education) were tested using the chi-square test for any significant difference between R1S2 and S1R2.

To appropriately analyze the crossover design, we followed the statistical framework outlined in [38] (pp. 7–29) and [39] (pp. 194–199) for all outcome measures (ADAS-Cog, NPI-Q, and MADRS). A mixed-effects model was implemented to evaluate the carry-over effect, period effect, and treatment effect, with participants included as the random effect. Following the mixed model analysis in [38,39], for participant $i(=1,\;2,\;\ldots ,n_{g})$ assigned to group g(=R1S2, S1R2), where $n_{g}$ is the total number of participants in group g, let $Y_{igp}^{\left(a\right)}$ denote the patient response at period p(=1, 2). We assume the following model:

(1) $$Y_{igp}=y_{igp}^{\left(a=2\right)}-y_{igp}^{\left(a=1\right)}$$

(2) $$Y_{igp}=\mu +\pi_{p}+\tau_{d[g,p]}+\gamma_{d[g,p-1]}+s_{ig}+\varepsilon_{igp}$$

where $y_{igp}^{\left(a\right)}$ denotes the measured outcome at either pre-treatment (a = 1) or post-treatment (a = 2), with larger values indicating an improved score; μ denotes the intercept; $\pi_{p}$ denotes the effect associated with the period; $\tau_{d[g,p]}$ denotes the effect associated with the treatment applied in period p of group g, where $d\left[g,p\right]$ is either the real or sham treatment; $\gamma_{d[g,p-1]}$ denotes the carry-over effect where it is assumed that $\gamma_{d[g,0]}=0$; $s_{ig}$ denotes the random effect associated with participant i in group g; and $\varepsilon_{igp}$ denotes the random errors with a mean of 0 and variance $\sigma_{e}^{2}$. To account for potential violations of model assumptions, such as heteroscedasticity or autocorrelation, the robust sandwich estimator is used. This method provides consistent estimates of the covariance matrix for parameter estimates even when the parametric model fails to hold. The model is implemented in SAS 9.4 M8 using the proc mixed procedure with the empirical option to apply robust standard errors and the Kenward–Roger degrees of freedom method. We did not perform separate preliminary tests for the period and carry-over effects as explained in [39] (pp. 39–44), as such tests lead to biased estimates of the direct treatment difference and increase the probability of Type I errors.

The hypotheses of this study were as follows:

1. Real tACS paired with cognitive exercises improves ADAS-Cog, NPI-Q, and MADRS scores in individuals with dementia more than sham stimulation $\left[\tau_{d[g,p]}\neq 0\right]$.
2. Both real tACS and sham tACS treatments, when combined with cognitive exercises, lead to improvements in ADAS-Cog, NPI-Q, and MADRS scores in individuals with dementia after a 4-week treatment period.
3. Real tACS will produce sustained improvements in ADAS-Cog, NPI-Q, and MADRS scores after the first treatment period compared to sham tACS. This will be observed by comparing the scores at the post-treatment assessment of the first period to the pre-treatment assessment of the second period.

To test the second hypothesis that both real and sham treatments would result in significant improvements in all outcome measures post-treatment compared to pre-treatment scores, we conducted one-sided paired *t*-tests separately for each treatment type. The significance threshold was adjusted to α = 0.025 following Bonferroni correction to account for multiple comparisons. To ensure there is no influence from the carry-over effect, only period 1 data were used.

Similar to our previous clinical trial [40], we used both ADAS-Cog and NPI-Q to classify the response rate of participants into four categories: “Marked”, “Moderate”, “No-Change”, and “Declining”. The criteria for classification are as follows (note that the AND is a logical and):

- Marked Response: a ≥ 3-point improvement in the ADAS-Cog score from pre- to post-treatment block.
- Moderate Response: an improvement in the ADAS-Cog score between 1 and 3 points AND an improvement or no change in the NPI-Q score.
- No-Change Response: a change in the ADAS-Cog score between −1 and 1 points OR an ADAS-Cog score improvement between 1 and 3 points AND a decline in the NPI-Q score.
- Declining Response: a decline of more than 1 point in the ADAS-Cog score.

The response rates were categorized into responders (combined “Marked” and “Moderate” responses) and non-responders (combined “No-Change” and “Declining” responses) due to limited samples. To assess treatment effects, we performed McNemar’s test, a statistical test for paired categorical data, by comparing response rates between the real tACS and sham conditions within subjects. Additionally, we reported descriptive statistics, including the percentages of each response type within each treatment group and period.

To evaluate the third hypothesis regarding the long-term effects of tACS compared to the sham, independent sample *t*-tests were used to compare changes in the outcome measures during the washout period. For this analysis, we calculated the change during the washout period by subtracting the post1 from the pre2 values, which served as the follow-up assessment. One-sided independent sample *t*-tests were then conducted to compare the calculated changes between the real tACS and sham tACS groups.

## 3. Results

**Figure 2:**
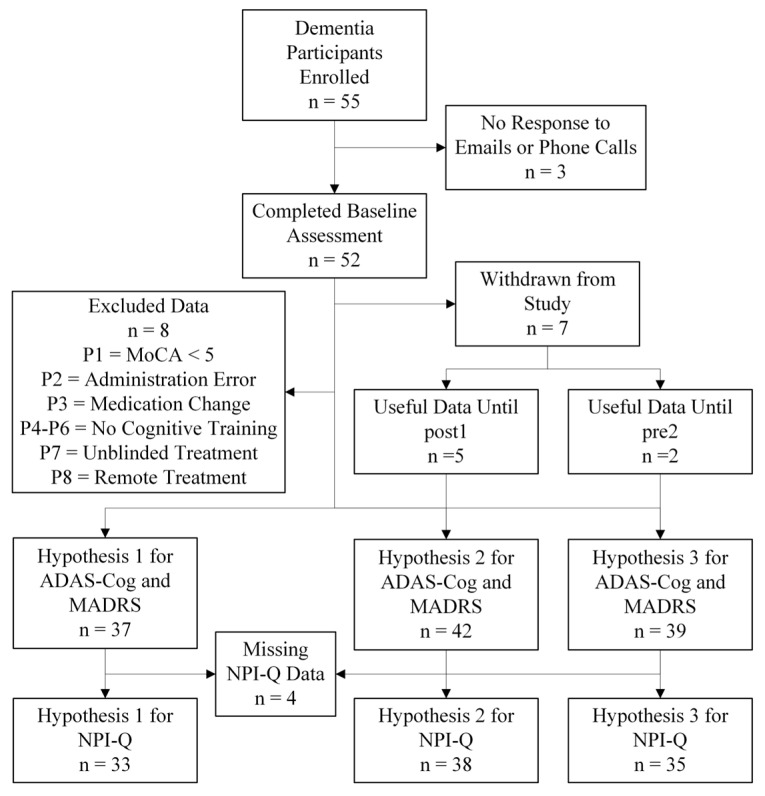
Flowchart of participant enrollment, including non-responders, baseline assessments, withdrawals, protocol breaches, and final analysis inclusion.

The study was conducted from 20 December 2021 to 31 July 2024, as originally planned, and concluded upon completion of the study period. A total of 55 participants with dementia were enrolled in the study. However, ten participants were lost to attrition, including seven who formally withdrew for various reasons, while three did not provide any reason. Of the seven withdrawn subjects, six had been assigned to R1S2 and one to S1R2 groups, resulting in five with usable data up to post1 or pre2. Table 1 lists the reasons for participants’ withdrawal and time of withdrawal. Furthermore, four participants had protocol breaches, which were excluded from the analysis; two participants were assigned to the R1S2 group, and two were in the S1R2 group. There were four participants whose data were excluded because (1) they live out of town and were not blinded; were (2) performing the treatment remotely, hence no tACS; and were (3) not compliant with the cognitive exercises. A flowchart of the enrolled participants and the participants excluded from the analysis is shown in Figure 2. The demographics for the 42 participants who were included in the analysis is shown in Table 2, including *p*-values comparing the baseline characteristics between R1S2 and S1R2. As shown, there were no significant differences between the two groups in terms of age, MoCA, MADRS, ADAS-Cog, NPI-Q, and sex, although education is marginally significant between R1S2 and S1R2. Importantly, no serious adverse effects were reported during this study. As for the washout period, while the majority followed the protocol of 8 weeks, some, due to unforeseen reasons, e.g., illness, emergencies, etc., had a longer washout period. The average washout duration for the R1S2 and S1R2 groups were 11.3 and 10.7, respectively.

### 3.1. ADAS-Cog Results

**Figure 3:**
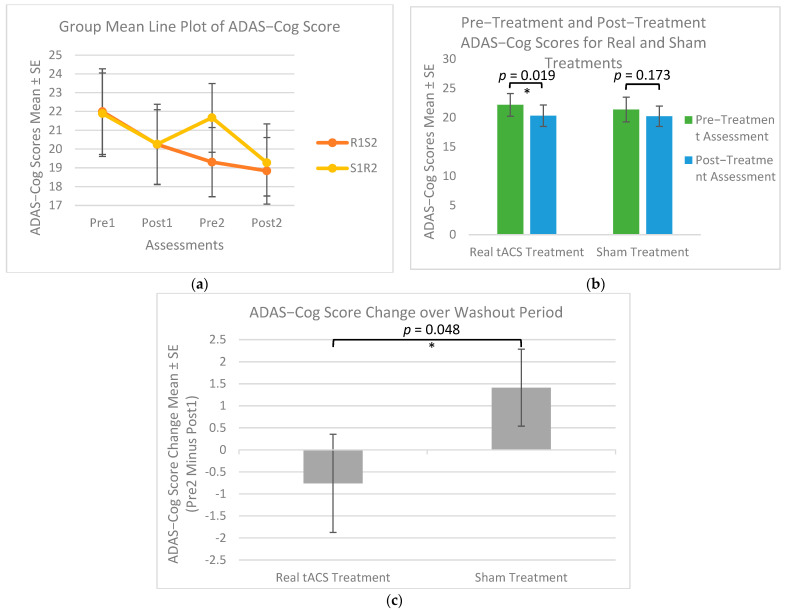

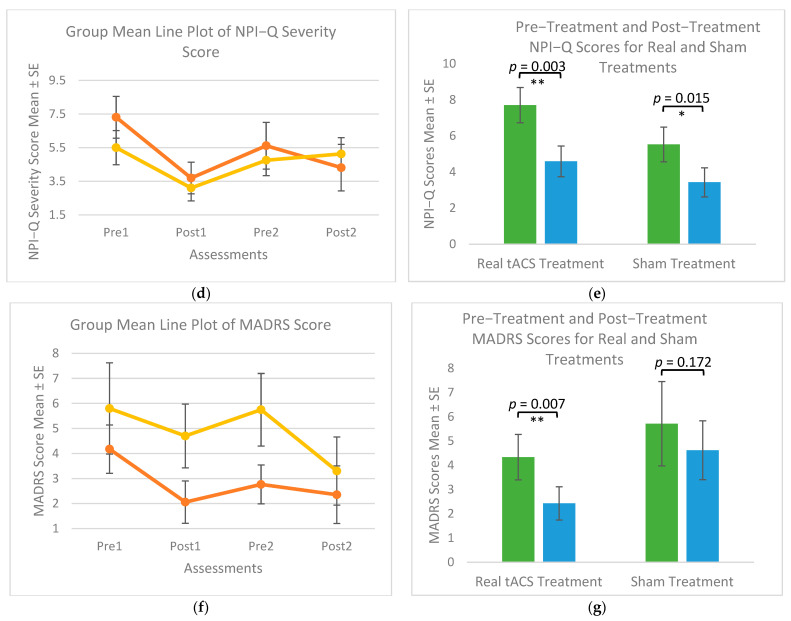
Comparison of cognitive and neuropsychiatric outcomes for real and sham tACS treatments. Lower scores for all outcome measures indicate improvements: (**a**) mean ADAS-Cog scores for each assessment, stratified by group; (**b**) ADAS-Cog score changes from pre-treatment to post-treatment in period 1; (**c**) ADAS-Cog score changes during the washout period; (**d**) mean NPI-Q presence scores for each assessment, stratified by group; (**e**) NPI-Q score changes from pre-treatment to post-treatment in period 1; (**f**) mean MADRS scores for each assessment, stratified by group; (**g**) MADRS score changes from pre-treatment to post-treatment in period 1. * *p*-value < 0.05 and ** *p*-value < 0.01.

The mixed-effects model analysis revealed no significant period effect (*p*-value = 0.633), carry-over effect (*p*-value = 0.414), or treatment effect (*p*-value = 0.934) observed on ADAS-Cog score differences (Table 3 and Table 4), indicating that real tACS did not result in greater cognitive improvements compared to sham treatment. Residual diagnostics confirmed model assumptions. The Anderson–Darling test showed no significant deviation from normality (A^2^ = 0.267, *p*-value *>* 0.250). Levene’s test indicated homogeneity of variances across periods (F = 0.09, *p*-value = 0.765). Residuals plotted against the order of data collection showed no discernible pattern, suggesting independence and no autocorrelation. Figure 3a illustrates the group means of ADAS-Cog scores for all four assessments, stratified by group.

Two paired samples *t*-tests were conducted to compare the ADAS-Cog scores before and after each treatment (real and sham) using data from period 1. To correct for multiple comparisons, a Bonferroni correction was applied, setting the adjusted significance threshold at α = 0.025. For the paired *t*-test for real tACS treatment, the assumption of normality was tested using the Shapiro–Wilk test on the difference in ADAS-Cog scores (*p*-value = 0.749). Outliers were assessed using Z-scores, and no outliers were detected. As shown in Figure 3b, there was a significant difference in the scores before treatment (M = 22.17, SD = 8.93) and after real tACS treatment (M = 20.32, SD = 8.45) (t(20) = 2.2183, *p*-value = 0.019). However, a paired samples *t*-test conducted to compare the ADAS-Cog scores before and after the sham tACS treatment showed no significant difference in the scores before (M = 21.37, SD = 9.71) and after treatment (M = 20.23, SD = 7.99) (t(20) = 0.9638, *p*-value = 0.173) (Figure 3b). Similarly, the Shapiro–Wilk test indicated normality (*p*-value = 0.883), and no outliers were detected using Z-scores. These results suggest that real tACS treatment significantly improved ADAS-Cog scores post-treatment, whereas sham treatment had no effect.

To analyze the long-term effects of tACS, an independent-sample *t*-test was used to compare changes in ADAS-cog scores during the washout period (from post1 to pre2) between the real and sham tACS groups. The pre2 assessment served as the follow-up reference point, allowing for a direct comparison of score changes. Prior to conducting the *t*-test, the Shapiro–Wilk test confirmed the normality of the data (*p*-value = 0.983 for the real treatment group and *p*-value = 0.522 for the sham treatment group), and Levene’s test verified the homogeneity of variances (*p*-value = 0.610). The results indicated a significant difference between the real tACS treatment (M = −0.76, SD = 4.32, *n* = 19) and sham tACS treatment (M = 1.41, SD = 3.60, *n* = 20) (t(37) = −1.709, *p*-value = 0.048) (Figure 3c), over the washout period. This suggests that real tACS had a significant effect on the improvement of ADAS-Cog scores during the washout period, while sham tACS did not produce a comparable change. These findings highlight the potential long-term benefits of real tACS in cognitive function beyond the immediate treatment phase.

### 3.2. NPI-Q Results

There were four participants from the R1S2 group who were missing NPI-Q scores due to either being unable to reach their caregivers or the assessor forgetting to administer the assessment.

The mixed-effects model analysis of the NPI-Q score differences revealed no significant treatment effects (*p*-value = 0.387) observed in the data (Table 5 and Table 6). However, there was a significant period effect (*p*-value = 0.003) and a marginally significant carry-over effect (*p*-value = 0.066) observed in the data. Residual diagnostics confirmed the homogeneity of variance and independence, and no autocorrelation was detected. The Anderson–Darling test showed significant deviation from normality (A^2^ = 1.080, *p*-value = 0.008); however, given the use of the robust sandwich estimator, the model remains valid despite this violation. Levene’s test indicated the homogeneity of variances across periods (F = 3.09, *p*-value = 0.084). Residuals plotted against the order of data collection showed no discernible pattern, suggesting independence and no autocorrelation. These findings highlight the importance of accounting for period and carry-over effects in crossover studies and indicate the limitations of NPI-Q, which are further discussed in the Discussion Section. Figure 3d illustrates the group means of NPI-Q scores for all four assessments, stratified by group.

Two paired samples *t*-tests were used to compare the NPI-Q scores before and after each treatment (real and sham). As shown in Figure 3e, there was a significant difference in the scores before treatment (M = 7.71, SD = 4.48) and after real tACS treatment (M = 4.59, SD = 3.91) (t(16) = 3.096, *p*-value = 0.003). The Shapiro–Wilk test indicated normality for the real (*p*-value = 0.084) and sham groups (*p*-value = 0.101), and no outliers were detected using Z-scores. The paired samples *t*-test conducted to compare the NPI-Q scores before and after the sham tACS treatment showed a significant difference in the scores of pre1 (M = 5.52, SD = 4.41) and post1 (M = 3.43, SD = 3.70) (t(20) = 2.3294, *p*-value = 0.015). These results suggest that both real and sham treatments had a significant effect on the neuropsychiatric symptom scores post-treatment compared to pre-treatment, with real tACS showing a greater effect.

A two-sample *t*-test was then used to compare changes in NPI-Q scores during the washout period, between the real tACS and sham tACS groups. Prior to conducting the t-test, the Shapiro–Wilk test confirmed the normality of the data (*p*-value = 0.842 for the real treatment group and *p*-value = 0.413 for the sham treatment group), and Levene’s test verified the homogeneity of variances (*p*-value = 0.946). The results indicated no significant difference between the real tACS treatment (M = 1.6, SD = 3.62, *n* = 15) and sham tACS treatment (M = 1.65, SD = 3.59, *n* = 20) (t(33) = −0.0406, *p*-value = 0.484). This suggests that there were no significant long-term effects of real tACS compared to sham tACS on neuropsychiatric symptoms during the washout period.

### 3.3. Response Rate

Table 7 presents the frequency of each response (Marked, Moderate, No-Change, and Declining) for both real and sham tACS treatment groups, separated by treatment periods (Period 1 and Period 2). To further evaluate the significance of changes in response rates, McNemar’s test was conducted to compare the proportion of responders (combined Marked and Moderate) between the real and sham tACS treatments. From the contingency table, adding Marked and Moderate responses for the real treatment shows that 57.7% of participants responded positively to real tACS, while adding Marked and Moderate responses for the sham treatment shows that 39.4% responded to sham tACS. The test revealed no statistically significant difference in response rates between real and sham tACS conditions (χ^2^(1) = 1.56, *p*-value = 0.211), indicating that response rates were comparable across conditions.

### 3.4. MADRS Results

The mixed-effects model analysis comparing real and sham tACS on MADRS scores revealed no significant carry-over effects (*p*-value = 0.644), period effects (*p*-value = 0.734), or treatment effect (*p*-value = 0.422) (Table 8 and Table 9). Residual diagnostics confirmed model assumptions. The Anderson–Darling test showed no significant deviation from normality (A^2^ = 0.47, *p*-value = 0.244). Levene’s test indicated the homogeneity of variances across periods (F = 0.15, *p*-value = 0.700). Residuals plotted against the order of data collection showed no discernible pattern, suggesting independence and no autocorrelation. These results suggest that real tACS did not show a significant effect compared to sham tACS on MADRS scores, indicating that neither treatment had a significant impact on depressive symptoms in this study. Figure 3f illustrates the group means of MADRS scores for all four assessments, stratified by group.

Wilcoxon signed-rank tests were used due to the Shapiro–Wilk test results (*p*-value *<* 0.03 for real and sham) to compare the MADRS scores before and after each treatment (real and sham). As shown in Figure 3g, there was a significant difference in the scores before (M = 4.33, SD = 4.29) and after real tACS treatment (M = 2.43, SD = 3.16); V = 81, *p*-value = 0.007. However, sham tACS treatment showed no difference in the scores before (M = 5.71, SD = 7.96) and after treatment (M = 4.62, SD = 5.57); V = 51.5, *p*-value = 0.172. These results suggest that real tACS treatment significantly improved depression scores post-treatment, whereas sham treatment did not show a significant effect.

A two-sample *t*-test was used to compare changes in MADRS scores during the washout period between the real tACS and sham tACS groups. The Shapiro–Wilk test confirmed the normality of the data (*p*-value = 0.918 for the real treatment group and *p*-value = 0. 111 for the sham treatment group), and Levene’s test verified the homogeneity of variances (*p*-value = 0.660). The results indicated a significant difference between the real tACS treatment (M = 1.16, SD = 3.44, *n* = 19) and sham tACS treatment (M = 1.05, SD = 4.08, *n* = 20) (t(37) = −0.089, *p*-value = 0.535). This suggests that there were no significant long-term effects of real tACS compared to sham tACS on depression during the washout period.

## 4. Discussion

In this study, we demonstrated that tACS paired with cognitive exercises significantly improves cognitive function with sustained effects lasting up to approximately 2–3 months compared to sham tACS with cognitive exercises (*p*-value = 0.048). The results align with findings from our previous pilot studies [6,7,8], which reported significant cognitive improvements with tACS paired with cognitive exercise compared to baseline. However, while the pilot study [8] showed a non-significant trend toward sustained cognitive effects of the real tACS group lasting up to 1 month after treatment, this study demonstrated statistically significant long-term effects lasting on average up to 2–3 months post-treatment. These differences could all be due to small sample numbers in our pilot studies compared to this study.

Our findings are mostly consistent with previous research on tACS in dementia. One study applying 40 Hz tACS over the left angular gyrus for 14 weeks showed a trend toward improvements at the end of the treatment period and toward sustained benefits lasting 2–3 months when measured using the Memory Index Score [10]. Another study applying 40 Hz tACS over the bitemporal lobes for 6 weeks found significant post-treatment improvements in the tACS group, whereas the sham group did not [9]. However, long-term effects differed, as ADAS-Cog scores in that study returned to baseline by the 12-week follow-up, while MMSE scores showed sustained improvement. Unlike these earlier studies, our results provide stronger evidence for the additive effects of tACS paired with structured cognitive training, showing sustained improvements 2–3 months post-treatment in ADAS-Cog scores.

The selection of the left DLPFC for stimulation was based on its central role in the default mode network, which is crucial for cognition, particularly memory consolidation and retrieval commonly impaired in dementia [41]. While there are many regions affected by dementia including the hippocampus and temporal lobe [42], there is evidence that the DLPFC is also significantly impacted. Structural and functional imaging studies have reported DLPFC’s involvement in AD [43,44], and preclinical work [45] supports this by showing how the prefrontal cortex layer 3 pyramidal cells displayed the lowest number of terminals. Given this, targeting the DLPFC with 40 Hz tACS may be particularly effective in modulating oscillatory dynamics relevant to cognitive dysfunction in dementia. Previous studies have demonstrated cognitive benefits when 40 Hz tACS is applied to the DLPFC [4,17]. Additionally, a sham-controlled study comparing 40 Hz tACS and tDCS over the DLPFC in individuals with MCI found that tACS led to significant improvements in the Stroop color test compared to tDCS, suggesting distinct cognitive benefits [46]. These findings reinforce the rationale for targeting the DLPFC in dementia research.

Both tACS and cognitive exercises have been shown to individually benefit cognitive function in individuals with dementia. However, their combination appears to amplify these effects. To date, our research team is the only one to have explored gamma-tACS paired with cognitive exercises [7,8]. Other studies have paired cognitive training with either theta-tACS or tDCS. For example, one study which applied theta-tACS every day for 1 week to individuals with MCI found significant improvements not only in the trained task but also in sustained attention, inhibitory control, and attentional orienting, thereby transferring benefits to untrained tasks [47]. However, they did not find improvements in working memory capacity, and those improvements were mostly seen in tasks related to the trained task. Another study applying theta-tACS, sham, or tDCS in healthy older adults for 10 sessions, twice weekly, found significant improvements in composite cognitive scores across all groups immediately post-intervention, with tDCS producing greater improvements in those with lower baseline MoCA scores [48]. The present study adds to this body of research by demonstrating the potential for gamma-tACS and cognitive exercise to produce lasting cognitive benefits.

A key in our study is that the daily cognitive exercises were not used as an evaluation tool, but rather as a means to cognitively challenge participants. Each session was guided by a trained treatment administrator, ensuring engagement and adherence to the exercises. Cognitive exercises assisted by a treatment administrator to instruct and help participants produced superior outcomes compared to unsupervised training, consistent with findings from [8,23]. This suggests that structured interventions with professional guidance enhance engagement and task-specific learning. It can be seen clearly from Figure 3a that the ADAS-Cog scores improved after 4 weeks of treatment, and both real tACS and sham tACS improved due to the cognitive exercises. However, the combination of tACS and cognitive training likely amplified these effects by concurrently modulating neural oscillations and activating task-relevant networks. When delivered during cognitive engagement, 40 Hz tACS may help entrain gamma oscillations in regions involved in the task, promoting more synchronized neural firing. This synchronization can enhance network efficiency and may support neuroplastic changes in circuits activated by cognitive exercises. Multiple, high-frequency sessions further reinforced these benefits, contributing to sustained cognitive improvements lasting up to approximately 2–3 months post-treatment, as seen in Figure 3a by the continued decline of ADAS-Cog scores from post1 to pre2 for the R1S2 group compared to the S1R2 group whose ADAS-Cog scores returned to their baseline score. As previously mentioned in the Introduction Section, previous studies have suggested that one of the effects of 40 Hz tACS is improving blood perfusion in the targeted area [11]. It is possible that repeated daily stimulation influenced blood flow dynamics, which may have contributed to the sustained cognitive improvements observed in the real tACS group compared to the sham. The analysis on the NPI-Q assessment, which measures neuropsychiatric symptoms, found a significant period effect and a marginally significant carry-over effect. This may be attributed to the nature of the assessment, as it relies on caregivers’ reports rather than participant self-assessments, with an assessor completing the final scoring. Caregivers’ responses may be influenced by factors such as emotional state, memory, expectations, or prior exposure to treatment [49]. The period effect suggests that caregivers may have formed assumptions about treatment efficacy based on their experiences in the first period. The analysis of the first-period data, due to the period effect, indicated a significant improvement in NPI-Q post-treatment compared to baseline for the real and sham groups. However, there was no significant difference in the long-term effects of either treatment on neuropsychiatric symptoms.

The response rates to treatment, based on ADAS-Cog and NPI-Q, were 57.7% for real tACS and 39.4% for sham treatment. This response rate is lower than in our previous clinical trial on repetitive transcranial magnetic stimulation treatments for AD [40]; however, in this study, we categorized “Responders” and “Non-responders” more strictly. Notably, we suspected that one non-responder had posterior cortical atrophy (PCA), a condition where a participant of a previous study was also not responsive to any brain stimulation. Another non-responder exhibited significant anxiety, which may have influenced cognitive performance.

Depression is often a confounding variable for cognitive improvement. It is important to note that among our participants, six had mild depression and two had moderate depression at baseline, as measured by MADRS. Depression is common in people living with dementia [50], and addressing mood disturbances is critical for improving the overall quality of life in this population. In the analysis of MADRS scores, we found a significant improvement in depression scores post-treatment compared to pre-treatment in the real tACS but not in the sham. However, neither treatment group had long-lasting effects on depression scores. These results suggest that the relationship between cognitive and depression scores may not be as strongly correlated as initially assumed. While cognitive scores improved post-treatment and continued to improve during the washout period for the real tACS group, MADRS scores did not follow the same pattern. The improvement in MADRS scores post-treatment may have been influenced by both tACS and the social interaction in the lab. However, the increase in MADRS score during the washout period (i.e., increased depression) may be due to the loss of social interaction or the possibility that tACS does not have long-lasting effects on depression. Contrary to our results, a meta-analysis on 40 Hz tACS found no significant improvement in depression scores post-treatment, but they stated that there were participants who reported improvements, suggesting that a larger sample size may find significant effects [51].

### Limitations

This study has several limitations that should be acknowledged. First, the relatively small sample size (*n* = 42), which is commonly seen in studies involving participants with dementia and brain stimulation, limits the generalizability of the findings. Recruitment challenges for this population, combined with the demanding protocol, made it difficult to recruit a larger sample. While our initial target was 60 participants, this number was not met by the study’s conclusion. Future studies with larger samples are necessary to validate these results and may provide information on whether mild dementia participants improve more compared to moderate dementia participants. Second, the study experienced a notable withdrawal rate of 18.2%, which may introduce selection bias and impact the reliability of the outcomes. Future research should explore ways to improve retention through enhanced participant support and reduced protocol burden. The third limitation is the variability in the washout period, which ranged from 8 weeks to 7 months. Initially, we aimed to maintain a fixed washout period, and most participants adhered to the 8-week schedule. However, numerous factors, including health issues, transportation difficulties due to the weather, and travel plans, hindered strict adherence to the study schedule, leading to variations in the washout period. This inconsistency impacted the analysis of long-term effects and limited our ability to accurately assess potential carry-over effects in the crossover design. Implementing a fixed or a washout period with less variability in future crossover trials may improve the interpretability of long-term outcomes. However, this approach may present a trade-off, as it could impact participant retention, particularly since individuals in this age group often travel for extended periods. Fourth, our participants had various dementia diagnoses including mixed dementia, AD, posterior cortical atrophy, vascular dementia, and frontotemporal dementia. Although the majority of participants had AD or mixed AD (62%), due to the variability in underlying pathology, progression rates, and responses to treatment, these different diagnoses may have introduced heterogeneity into the results, making it difficult to generalize the findings. Our decision to include a broader range of dementia diagnoses was driven by the need to recruit enough participants to approach the original sample size calculation. While this approach aimed to enhance the statistical power of the study, it introduced variability in the dementia types, which complicates the interpretation of the results. Recruiting enough participants to stratify them based on their specific dementia diagnosis and ensuring an adequate sample size are very challenging. However, future research should consider addressing these challenges to better understand the differential effects of the intervention. Conducting subgroup analyses may help determine whether certain types of dementia respond better to the intervention compared to others. Fifth, the potential practice effect with ADAS-Cog must be considered, as repeated exposure to the same cognitive tests could lead to improved scores unrelated to the intervention itself [52]. Our attempt to control for practice effect was to use alternate versions of ADAS-Cog that use different word lists. Nevertheless, the remaining tests are the same across the assessments. Additionally, the influence of participants’ mood on the day of their assessment will influence their test result. However, it is very difficult to control for a participant’s mood and find a way to mitigate this effect, but having a large sample size can help overcome this confounding variable. Addressing these limitations in future research will be crucial to refining the study design and enhancing the reliability of the findings.

## 5. Conclusions

This study provides evidence that 4 consecutive weeks of gamma-frequency transcranial alternating current stimulation (tACS) paired with cognitive exercises can significantly improve cognition, neuropsychiatric symptoms, and depression in individuals with dementia post-treatment compared to pre-treatment. In this study, we demonstrated that real tACS not only improves cognitive performance post-treatment but also produces sustained benefits compared to sham stimulation. The findings from this study highlight the significance of combining tACS and cognitive exercise as a promising non-invasive treatment for dementia and related cognitive disorders.

## Figures and Tables

**Table 1:** Reasons for participant withdrawal in the clinical trial.

Participant	Group	Withdrawal Reason	Time of Withdrawal
P008	R1S2	Personal issues	After 4 treatment days in the first period
P012	R1S2	Difficulty attending the treatments due to health decline	After pre2 assessment
P016	R1S2	Moving to a long-term care facility	After 14 treatment days in the second period
P021	R1S2	Difficulty attending the treatments due to health decline	After post1 assessment
P037	S1R2	Participant and their caregiver perceived the study as too long	After post1 assessment
P039	R1S2	Did not want to participant and continued health decline	After post 1 assessment
P046	R1S2	Hesitant to start treatment	After 9 treatment days in the first period

Note: Pre2 assessment is the assessment before the second period. Post1 assessment is the post-treatment assessment of the first period.

**Table 2:** Descriptive statistics summarizing the demographic characteristics of study participants stratified by group.

Participant Characteristic	R1S2 Group (*n* = 21)	S1R2 Group (*n* = 21)	*p*-Value
Age (years)	72.9 ± 8.6	76.3 ± 9.3	0.227
MoCA	16 ± 5.8	16 ± 5.1	0.955
MADRS	4.3 ± 4.3	5.7 ± 8.0	0.969
ADAS-Cog	22.2 ± 8.9	21.4 ± 9.7	0.783
NPI-Q	7.7 ± 4.5	5.5 ± 4.4	0.141
	(*n* = 17)	(*n* = 21)	
Sex			
Female	10 (47.6%)	6 (28.6%)	0.341
Male	11 (52.4%)	15 (71.4%)	
Handedness			
Right	18 (85.7%)	20 (95.2%)	
Left	3 (14.3%)	0	
Ambidextrous	0	1(4.8%)	
Education			
High school or below	9 (42.9%)	10 (47.6%)	0.133
Above a high school diploma	12 (57.1%)	11 (52.4%)	

Note: The *p*-values correspond to the appropriate independent t-test, Mann–Whitney U test, or Chi-squared test to compare R1S2 and S1R2 group baseline characteristics. Continuous variables are presented as the mean ± SD and categorical variables as *n* (*%*). Abbreviations: Montreal Cognitive Assessment (MoCA), Montgomery–Åsberg Depression Rating Scale (MADRS), Alzheimer’s Disease Assessment Scale–Cognitive subscale (ADAS-Cog), and Neuropsychiatric Inventory Questionnaire (NPI-Q).

**Table 3:** Fixed effect estimates from the mixed-effects model analysis for ADAS-Cog score differences, including carry-over, period, and treatment (real vs. sham) effects.

Effect	Estimate	Standard Error	DF	t Value	*p*-Value
Intercept	−0.4635	0.8841	35	−0.52	0.6034
Carry-over (no)	−1.8178	2.2005	35	−0.83	0.4144
Period (1)	0.6519	1.3514	35	0.48	0.6326
Treatment (Real)	−0.1181	1.4140	35	−0.08	0.9339

**Table 4:** Type 3 tests of fixed effects in the mixed-effects model analysis for ADAS-Cog score differences.

Effect	Num DF	Den DF	F Value	*p*-Value
Period	1	35	0.23	0.6326
Treatment	1	35	0.01	0.9339
Carry-over	1	35	0.68	0.4144

**Table 5:** Fixed effect estimates from the mixed-effects model analysis for NPI-Q score differences, including carry-over, period, and treatment (real vs. sham) effects.

Effect	Estimate	Standard Error	DF	t Value	*p*-Value
Intercept	−1.3077	0.5031	31	−2.60	0.0142 *
Carry-over (no)	2.8981	1.5208	31	1.91	0.0660
Period (1)	−3.9904	1.2539	31	−3.18	0.0033 **
Treatment (Real)	−1.2154	1.3850	31	−0.88	0.3869

Note: * *p*-value < 0.05, ** *p*-value < 0.01.

**Table 6:** Type 3 tests of fixed effects in the mixed-effects model analysis for NPI-Q score differences.

Effect	Num DF	Den DF	F Value	*p*-Value
Period	1	35	10.13	0.0033 **
Treatment	1	35	0.77	0.3869
Carry-over	1	35	3.63	0.0660

Note: ** *p*-value < 0.01.

**Table 7:** Frequency of the different response types in each treatment and each period.

Group	Period	Treatment	Marked Response	Moderate Response	No-Change Response	Declining Response
R1S2	1	Real	5 (15.2%)	2 (6.1%)	3 (9.1%)	3 (9.1%)
	2	Sham	4 (12.1%)	0	5 (15.2%)	4 (12.1%)
S1R2	1	Sham	7 (21.2%)	2 (6.1%)	6 (18.2%)	5 (15.2%)
	2	Real	9 (27.3%)	3 (9.1%)	4 (12.1%)	4 (12.1%)

Note: The values are written as *n* (%) where the percentage is calculated out of the total number of participants.

**Table 8:** Fixed effect estimates from the mixed-effects model analysis for MADRS score differences, including carry-over, period, and treatment (real vs. sham) effects.

Effect	Estimate	Standard Error	DF	t Value	*p*-Value
Intercept	−0.4118	0.9524	35	−0.43	0.6681
Carry-over (no)	−1.0206	2.1898	35	−0.47	0.6441
Period (1)	0.3324	0.9693	35	0.34	0.7337
Treatment (Real)	−1.0176	1.2536	35	−0.81	0.4224

**Table 9:** Type 3 tests of fixed effects in the mixed-effects model analysis for MADRS score differences.

Effect	Num DF	Den DF	F Value	*p*-Value
Period	1	35	0.12	0.7337
Treatment	1	35	0.66	0.4224
Carry-over	1	35	0.22	0.6441

## Data Availability

The data that support the findings of this study are available from the corresponding author upon reasonable request.

## Abbreviations

The following abbreviations are used in this manuscript:

AD	Alzheimer’s Disease
ADAS-Cog	Alzheimer’s Disease Assessment Scale—Cognitive subscale
DLPFC	Dorsolateral Prefrontal Cortex
MADRS	Montgomery–Åsberg Depression Rating Scale
MCI	Mild Cognitive Impairment
MMSE	Mini-Mental State Examination
MoCA	Montreal Cognitive Assessment
NPI-Q	Neuropsychiatric Inventory Questionnaire
tACS	Transcranial Alternating Current Stimulation
tDCS	Transcranial Direct Current Stimulation
WMS	Wechsler Memory Scale
